# Pilin regions that select for the small RNA phages in *Pseudomonas aeruginosa* type IV pilus

**DOI:** 10.1128/jvi.01949-24

**Published:** 2025-02-27

**Authors:** Hee-Won Bae, Hyeong-Jun Ki, Shin-Yae Choi, You-Hee Cho

**Affiliations:** 1 Program of Biopharmaceutical Science, Department of Pharmacy, College of Pharmacy and Institute of Pharmaceutical Sciences, CHA University, Seongnam-si, Gyeonggi-do, South Korea; Michigan State University, East Lansing, Michigan, USA

**Keywords:** *Pseudomonas aeruginosa*, fiersphage, type IV pilus, pilin, phage selection

## Abstract

Type IV pili (TFPs) serve as the primary receptor for phages in *Pseudomonas aeruginosa* (PA), playing a key role in phage selection and determining the phage host range. We previously demonstrated that a fiersphage *Pepevirus rubrum* (PP7) selectively infects PA strains with group II (G2) TFP pilin. In this study, we expanded on this by profiling the host range of another fiersphage, *Pepevirus spumicola* (LeviOr01), which was able to infect several PA strains including PMM23. Analysis of 47 in-house PA strains showed that PMM23, along with nine other LeviOr01-susceptible strains and four resistant strains, possessed group I (G1) TFP pilin. This suggests that LeviOr01 targets specific regions of G1 pilin for phage-receptor interaction. Comparative sequence analysis revealed significant differences in the amino acid composition of the αβ and β1-β2 loop regions between susceptible and resistant G1-pilin strains. Pilin switching experiments using a PA surrogate strain (PAK) lacking endogenous pilin confirmed that G1 pilin is essential for LeviOr01 infection, while the accessory gene (*tfpO*) associated with G1 pilin was not involved in phage susceptibility. Structural predictions and comparisons between G1 and G2 pilins revealed conformational similarities but distinct differences at the αβ loop, which is likely critical for interacting with the phage virions. These findings suggest that the αβ loop region plays a pivotal role in determining RNA phage selectivity, offering new insights into the molecular mechanisms governing phage-host interactions and the host tropism of the RNA phages in various PA strains.

**IMPORTANCE:**

The host range of bacteriophages (phages) is crucial for both fundamental research and practical applications, particularly when targeting bacterial pathogens like *Pseudomonas aeruginosa* (PA). Previous studies have shown that the RNA phage PP7 (*Pepevirus rubrum*) binds to the αβ loop region of group II (G2) pilin. In this study, we demonstrate that another RNA phage, LeviOr01 (*Pepevirus spumicola*), also relies on the same region in group I (G1) pilins for infection. Computational modeling and structural comparisons between G1 and G2 pilins suggest that variations in the αβ loop region determine the selectivity of RNA phage binding, with similar interactions observed in both pilin groups. These findings enhance our understanding of the molecular interactions between RNA phages and their pilin receptors, offering valuable insights for developing RNA phage-based therapeutic strategies to combat PA infections.

## INTRODUCTION

The host range of a phage refers to the spectrum of bacterial hosts that can support the phage life cycle by providing essential resources. This range is primarily shaped by the interaction between bacterial defense and phage adaptation that enables them to overcome these barriers. One of the key factors determining phage host range is receptor recognition, as this interaction occurs at the earliest stage of infection and dictates the specificity of the phage for its bacterial host (1). Bacteria present a variety of surface receptors, such as cell wall components and cellular appendages, which differ across strains and species. This natural variability often limits the phage host range to those bacteria with compatible receptors (2). Surface receptors can be chemically modified by specific enzymes to interfere with the phage interaction with the receptors and/or physically masked by extracellular or capsular polysaccharides to obstruct the phage access to the receptors (3).

*Pseudomonas aeruginosa* (PA), a versatile and opportunistic pathogen, is one of the bacteria showing extreme genetic diversity. Analysis of genome sequences from 7,982 PA strains, available in the *Pseudomonas* Genome Database (http://pseudomonas.com), has revealed that PA harbors a rich repertoire of defense and resistance genes against phages, though the exact mechanisms remain unclear (4, 5). Like in many other bacteria, the primary phage receptors in PA are lipopolysaccharides (cell wall components) and type IV pili (TFPs) (cellular appendages). TFPs, in particular, serve as crucial receptors for numerous PA phages, including RNA phages of the *Fiersviridae* family (6, 7).

Our previous work demonstrated that the fiersphage *Pepevirus rubrum* (PP7) binds to group II (G2) TFP pilins, with the variable region of the pilin being a key determinant for PP7 adsorption (8). This was confirmed by a structural study of the PP7-TFP complex, which identified four key amino acids (K65, I68, E77, and Y79) in the αβ loop region as critical for PP7-TFP interaction (9). Since TFP pilins are conserved across all PA strains, the variability in these pilins could be an important determinant of the host range for TFP-binding fiersphages. However, due to the significant genetic diversity in PA pilin regions, the precise molecular mechanisms by which pilin variability affects fiersphage host specificity remain poorly understood.

In this study, we investigate the role of TFP pilin variability in determining the host range of fiersphages in PA strains. We profiled the host range of another fiersphage, *Pepevirus spumicola* (LeviOr01), to further explore the interaction between RNA phages and TFP pilins. By combining pilin switching experiments and structural modeling, we confirmed the involvement of key amino acid residues in the αβ loop region, which are crucial for phage recognition and selection. These findings offer new insights into the molecular determinants of phage-host specificity in PA strains.

## RESULTS AND DISCUSSION

### Pilin grouping and RNA phage susceptibility

Our previous research demonstrated that certain group II (G2) type IV pilins (TFPs) are essential for infection by the fiersphage *Pepevirus rubrum* (PP7) (8). In addition to PP7, another fiersphage that is known to infect *Pseudomonas aeruginosa* (PA) strains is *Pepevirus spumicola* (LeviOr01) (10). In contrast, LeviOr01 is known to infect certain clinical PA strains, such as PcyII-10, whose genome was recently sequenced (accession number LT673656). Given that LeviOr01 does not infect commonly used PA strains like PAO1 and PA14 and that PcyII-10 contains a group I (G1) pilin associated with the *tfpO* gene (Fig. S1), we hypothesized that LeviOr01 requires G1 pilins for infection. To explore this, we first evaluated LeviOr01 susceptibility and determined the pilin grouping of 47 PA strains from our collection (8, 11). As shown in Fig. S1A, some G1 pilin strains were clearly susceptible to LeviOr01, while some G2 pilin strains were susceptible to PP7. This result suggests that LeviOr01 specifically targets G1 pilins, while PP7 favors G2 pilins, indicating that different RNA phages select distinct pilin groups for infection. To further explore the pilin determinants that influence RNA phage host tropism in PA strains, we performed a multiple sequence alignment of pilins from the 47 strains and PcyII-10, generating a phylogenetic tree (Fig. S1B). Four out of 10 group III (G3) pilins clustered together in the first clade, suggesting that G3 pilins are the most closely related to ancestral pilins. The tree also suggests that G1 pilins evolved from G2 pilins. This complex evolutionary landscape warrants further investigation, especially since G2 pilins lack accessory genes, whereas G3 and G1 pilins possess different accessory proteins: G3 pilins are associated with the minor pilin component (TfpY), while G1 pilins are linked to the glycosylase enzyme (TfpO) (12). Notably, LeviOr01-resistant G1 pilin strains, such as PMM33, clustered together, while PP7-sensitive G2 pilins, including PAO1 and PMM49, formed a closely related subgroup. These results suggest that phage-resistant G1 pilin strains likely evolved later to resist LeviOr01, while phage-sensitive G2 pilin strains emerged later for PP7. As with our study on PP7, this investigation provides a foundation for identifying the specific pilin regions that determine LeviOr01 susceptibility through sequence comparison of G1 pilins.

### G1 pilin expression in the surrogate strain

**Fig 1:**
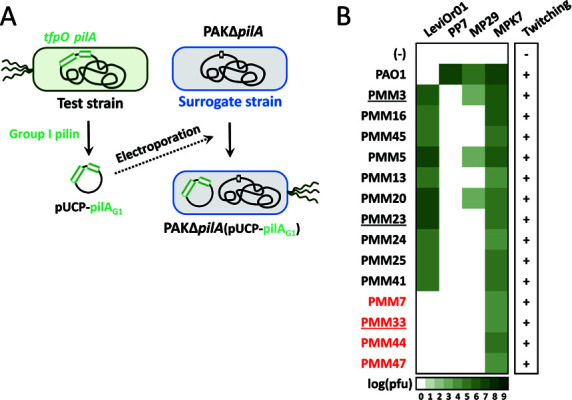
Pilin switching experiments. (**A**) Schematic representation of pilin switching experiments. The pilin regions containing both the *tfpO* and *pilA* genes of G1-pilin test strains were cloned into the plasmid pUCP18 (pUCP-pilA_G1_) and then introduced into the surrogate strain, which is an in-frame deletion mutant of the *pilA* gene in the PAK strain background (8). The resulting PAK-derivative strains expressing G1 pilins were assessed for phage susceptibility and twitching motility in (B). (**B)** Heat map displaying the relative efficiency of plaque formation (EOP) for LeviOr01. The color gradient represents the relative log(pfu) values, as indicated at the bottom. Those for the DNA phages (MP29 and MPK7) are also depicted as controls. The values are the averages of three biological replicates. Strains resistant to LeviOr01 are marked in red. The twitching motility status for each strain is shown on the right of the heat map: +, twitching-proficient; −, twitching-defective. Three G1 pilins analyzed further are underlined (PMM3, PMM23, and PMM33). The *pilA* region from PAO1 was introduced as well as a control.

We noted that certain strains (e.g., PMM13 for LeviOr01 and PMM34 and PMM37 for PP7), despite clustering with susceptible strains, were twitching-defective and resistant to other TFP-dependent DNA phages (MP29 and MPK7) (Fig. S1A). To confirm the connection between LeviOr01 host tropism and pilin variability, we established a surrogate system for pilin switching using a PAK *pilA* mutant (Fig. 1A). This approach was based on our previous findings that RNA phage propagation is most efficient in PAK (6, 8, 13) and that MPK7 could serve as a control (14). The twitching motility and MPK7 susceptibility were fully restored by the G1 pilin region that encompasses both *tfpO* and *pilA* genes from the G1-pilin strains (Fig. 1B). PP7 susceptibility was mediated by the PAO1 pilin, while LeviOr01 susceptibility was restored by the pilin regions from the susceptible strains, except for PMM13. Since the PMM13 pilin is clustered with those of the susceptible strains (Fig. S1B) and PMM13 was defective in twitching motility (Fig. S1B), we suggest that LeviOr01 resistance of PMM13 was due to TFP dysfunction, rather than pilin variability. Thus, pilin variability appears to be the primary barrier for both fiersphages, PP7 and LeviOr01.

### Sequence comparison of G1 and G2 pilins

**Fig 2:**
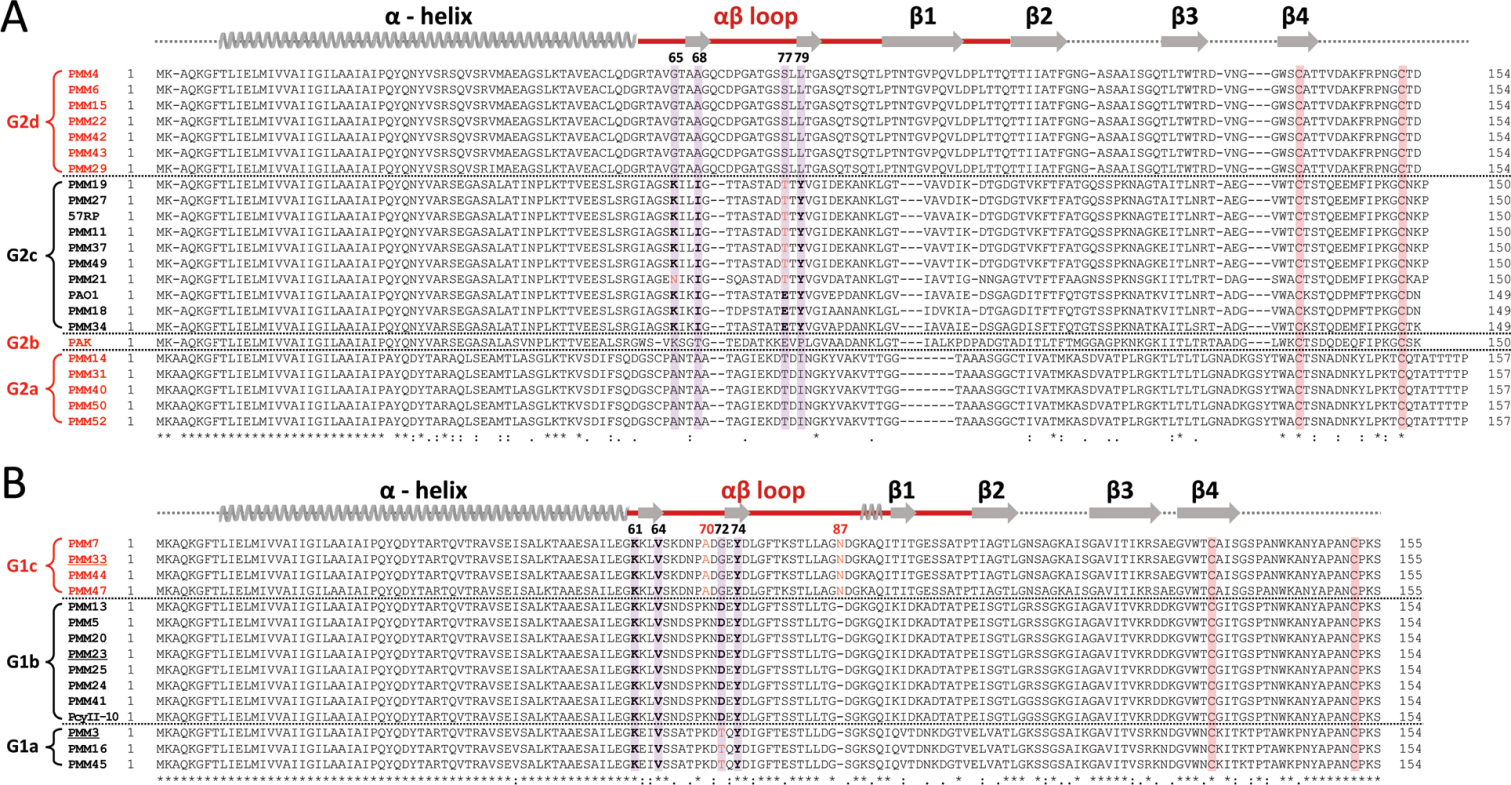
Multiple sequence alignment of G2 and G1 pilins. Multiple sequence alignment of G2 (**A**) and G1 (**B**) pilins performed using the Clustal X program. Twenty-three G2 pilins from four clusters (G2a to G2d) and 15 G1 pilins from three clusters (G1a to G1c) are as depicted in Fig. S1B, separated by dashed lines. LeviOr01-resistant and PP7-resistant strains are highlighted in red. Identical and homologous residues are indicated by * and :, respectively. Conserved secondary structures among TFP pilins are depicted above the sequences. Regions with the greatest variability between the pilins are outlined in red lines, corresponding to the αβ and β1-β2 loop regions. The four key residues involved in PP7 adsorption (**A**) and those corresponding to G1 pilins (**B**) are highlighted in bold and purple shade with the residue numbers. Additional notable amino acids are indicated in red (see texts). Two cysteine (C) residues for the C-terminal variable loop are shaded in pink. The three G1 pilins used in further analysis are underlined (PMM3, PMM23, and PMM33).

Our previous research demonstrated that the β1-β2 loop region influences PP7 susceptibility, which varies significantly between susceptible and resistant G2 pilins (8). The cryo-electron microscopy (EM) structure of both the PAO1 TFP and the PAO1 TFP-PP7 complex showed that the overall negative charge of the αβ loop region in PAO1 pilin supports electrostatic interactions with PP7 maturation protein (MP) at the positively charged minor β-sheet region (9) (Fig. S2). This study also identified four key residues (K65, I68, E77, and Y79) that could determine PP7 selectivity in PAO1 pilin. A multiple alignment of G2 pilins grouped them into four clusters (G2a to G2d) (Fig. 2A), with 10 PP7-susceptible pilins found in the G2c cluster. However, the four key residues are conserved in only three G2c pilins, while the other seven have T77 instead of E77, and PMM21 pilin contains N65 instead of K65. These findings align with the cryo-EM data and also suggest that structural variation at the β1-β2 loop region most likely influences the overall conformation of the αβ loop region, affecting its interaction with PP7 virions.

We next investigated whether sequence or structural similarities between the pilins could explain RNA phage-TFP interactions. Following the cryo-EM data from Thongchol et al. (9), we updated our pilin numbering scheme by adding six to each residue from our previous study, which had used the numbering by Hartung et al. (15). In their study, they truncated the N-terminal six amino acids for the PAK pilin (PDB code 1OQW). Figure 2B shows that G1 pilins have similar overall sequences, with variable regions after the N-terminal core α-helix. Notably, the αβ loop region contains two short β-sheets, as seen in G2 pilins, and four residues (K61, V64, D72, and Y74) are similarly located, suggesting that the interaction landscape between LeviOr01 virions and TFP receptors may not differ substantially from that for PP7.

### Structural modeling of group I pilins

**Fig 3:**
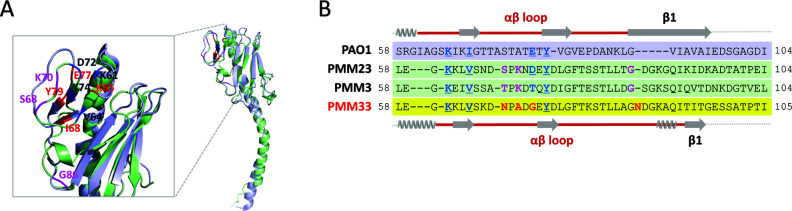
Structural modeling of key residues in G1 pilin. **(A)** Structural overlay of PAO1 (**G2**) and PMM23 (**G1**) pilins. The solved structure of the G2 pilin (purple) was superimposed on the AlphaFold2-predicted structure of the G1 pilin (green) highlighting overall structural variation. The αβ loop regions, containing the four key residues (red for G2 and blue for G1), are shown on the left. The residues for mutational analysis (S68, K70, and G86) for PMM23 pilin are indicated in pink (see Fig. 4). (**B)** Sequence comparison of PAO1 (**G2**) and PMM23 (**G1**) pilins at the variable region. A partial sequence alignment of PAO1 and three G1 pilins is displayed as in Fig. 2. G1a (PMM3) and G1b (PMM23) pilins are susceptible to LeviOr01, while G1c (PMM33) pilin is resistant (red). Secondary structures are shown above (PAO1) and below (**G1**) the sequences. The αβ loop regions are outlined in red. The four key residues for RNA phage interactions are in boldface and underlined, with notable amino acids in PMM33 highlighted in red. The residues for mutational analysis (S68, K70, and G86) for PMM23 pilin are indicated in pink (see Fig. 4).

To explore whether RNA phage-TFP interactions are influenced by structural contexts, we performed AlphaFold2 modeling of PMM23 pilin and compared it to the cryo-EM structure of PAO1 pilin. The superimposition of the predicted structure of the PMM23 pilin monomer and the cryo-EM-based structure of the PAO1 pilin monomer (Fig. 3A) shows that structural differences are most prominent at the αβ and β1-β2 loop regions. These differences in the configuration of four key residues may explain pilin-specific selection of the RNA phages.

Further analysis of the αβ and β1-β2 loop sequences in G1 pilins from PMM3 (LeviOr01-sensitive G1a), PMM23 (LeviOr01-sensitive G1b), and PMM33 (LeviOr01-resistant G1c) suggests that specific sub-regions determine RNA phage selection in the G1 pilins for LeviOr01 in analogy with PAO1 pilin for PP7. As shown in Fig. 2B and 3B, some amino acids are different between susceptible (i.e., PMM3 and PMM23) and resistant (i.e., PMM33) pilins at the αβ loop region: two conserved residues unique to G1c pilins, the 70th alanine (A70) and the 87th asparagine (N87), likely contribute to LeviOr01 resistance. However, it is more plausible that the overall structural organization around these residues at the αβ loop is responsible for LeviOr01 resistance in G1c pilins.

### Key residues in pilin for RNA phage selection

To identify critical amino acid residues for LeviOr01 selection, we created mutant PMM23 pilins with amino acid changes specific to the LeviOr01-resistant PMM33 pilin (Fig. 2B and 3B). Furthermore, we additionally evaluated the involvement of TfpO, the canonical accessory protein for G1 pilins in LeviOr01 susceptibility. It is known that TfpO glycosylates each pilin of TFP with one subunit of *O*-antigen and confers resistance to opsonization directed by the pulmonary surfactant protein A during host-mediated phagocytosis (16). It would be intriguing if TfpO could also affect the phage susceptibility, suggesting the multiple roles of this glycosylase in the survival of PA strains.

Using the surrogate system with episomal pilin expression, we simply generated the mutations in the wild-type PMM23 pilin and then the mutant pilin-expressing plasmids were introduced into the surrogate strain. Two PAO1 mutant pilins (I68S and Y79D) were also created to confirm the effect of the key residues in PP7 susceptibility. These two were chosen among the four key amino acid residues, because they are perfectly conserved among the 10 G2c pilins, unlike the other two amino acids (K65 and E77) (Fig. 2A).

**Fig 4:**
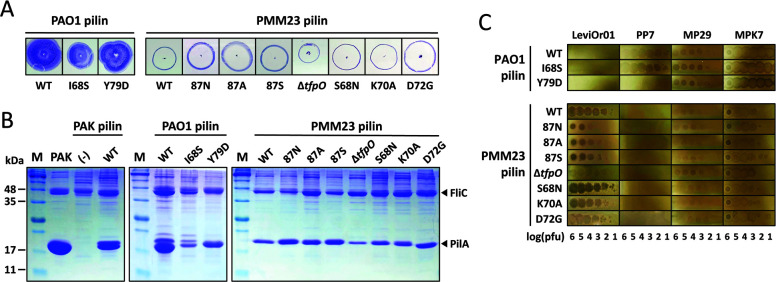
Mutational analysis of pilin specificity for RNA phages. (**A)** Twitching motility phenotypes. The surrogate strains expressing PAO1 or PMM23 pilin regions with designated mutations were subjected to twitching motility tests. Substitution (I68S and Y79D) mutants in PAO1 pilin and substitution (S68N, K70A, and D72G for PMM23 pilin), in-frame deletion (Δ*tfpO*), and insertion (87N for G86_D87insN, 87A for G86_D87insA, and 87S for G86_D87insS) mutants in PMM23 pilin were analyzed alongside wild-type (WT) PAO1 and PMM23 pilins. Photographs of the twitching test plates were taken after 24 h. (**B)** Surface piliation. Surface proteins from the strains indicated as in (**A)** were analyzed by SDS-PAGE, followed by staining. Flagellin (FliC) and pilin (PilA) bands are marked by arrowheads. Molecular weight markers (M) indicate 11, 17, 35, and 48 kDa bands. (**C)** Phage susceptibility. Serial dilutions of phage lysates were spotted onto the same strains in (**A)**, with the numbers indicating the log(pfu) of phage spots. TFP-requiring DNA phages, MP29 and MPK7, were used as control.

Figure 4A shows the twitching motility phenotypes of the surrogate strains with the mutant pilins. The twitching patterns of the surrogate strains harboring the PAO1 pilins are different from those with the PMM23 pilins. Despite a slight decrease in twitching diameter of the surrogate cells with the PAO1(I68S) and the PMM23(Δ*tfpO*) pilins, most of the mutant pilins did not differ from the cognate wild-type pilins regarding the pattern and size of the twitching zone. Since PAK is a highly invasive PA strain (17) and it was relatively hyperpiliated so that the surface piliation was easily observed just by Coomassie staining after gel separation of the surface proteins (Fig. 4B), the pilin bands were verified by the plasmid-borne expression of the pilin region of the mutant pilins. No significant mobility change was observed for the *tfpO* in-frame deletion mutant (Δ*tfpO*), only with somewhat lower intensity of the pilin, suggesting that TfpO might affect the stability of the PMM23 pilin in the PAK background, which needs to be further verified.

We next tested the mutant-pilin bacteria for their adsorption to LeviOr01 and susceptibility to TFP-requiring phages as in Fig. S1A and B, Fig. S3 ad S4C). While PAO1(Y79D) pilin rendered cells resistant to PP7, PAO1(I68S) pilin did not, confirming the critical contribution of Y79 in RNA phage selection. It was clear that neither of the two mutations significantly altered the susceptibility to TFP-requiring DNA phages despite a minute decrease in plaque formation efficiency for MP29 on the cells with PAO1(Y79D). This result substantiates the critical but differential contribution of the four key residues in the direct interaction with PP7 MP. Among the PMM23 mutants, only the PMM23(S68N) mutant retained adsorption and susceptibility to LeviOr01, while insertion of asparagine (N) at the 87th position (G86_D87insN or 87N) reduced LevOr01 adsorption and susceptibility, which was also observed for the insertion of alanine (A) and serine (S) (i.e., in 87A and 87S) as well. The other two substitution mutants (K70A and D72G) also rendered the cells slightly less susceptible to LeviOr01 with the concomitant LeviOr01 adsorption. It should be noted that the root mean square deviation (RMSD) from AlphaFold3-based structural superimposition between the mutant and PMM23 pilins shows some correlation with adsorption and susceptibility to LeviOr01 (Fig. S3). This suggests that the overall conformation of the αβ loop region plays a critical role in RNA phage selection in both G1 and G2 pilins.

### Conclusions

Based on our previous study showing that the RNA phage PP7 (*Pepevirus rubrum*) interacts specifically with a set of group II (i.e., G2c) pilins for infection (8), we have suggested that the sequence variation around the β1-β2 loop of G2 pilins could be a determinant of the host spectrum of this RNA phage. Recent cryo-EM studies about the structure of TFP and the PP7-TFP complex confirmed that PP7 MP binds to a single pilin subunit in the TFP, where four key residues of the PAO1 (G2c) pilin (K65, I68, E77, and Y79) are involved in the interaction with the minor β-sheet of MP (9). Here we expanded our understanding of the pilin determinants of the host spectrum for the small RNA phages by profiling the host range of another RNA phage, LeviOr01 (*Pepevirus spumicola*), and demonstrating that the structural contexts at the αβ loop region are the determinants for the host tropism of the RNA phages in PA, irrespective of the accessory proteins in pilin variability.

An interesting avenue for future study would be to explore RNA phage selection by other types of pilins such as IncF and IncP pilins. For example, another well-known RNA phage, PRR1 (*Perrunavirus olsenii*), would be the next candidate because it requires the conjugative IncP pilus encoded by RK2 (RP4), which is widely distributed among Gram-negative bacteria (18). This research not only enhances our understanding of the underappreciated ecology of RNA phages as well as the molecular basis of the interactions between the pilin and the RNA phage MP but also sheds light on the principle for the host spectrum of the RNA phages in adsorbing to the susceptible pilins as their receptor. Additionally, elucidating these interactions can inform the design of RNA phage-based therapeutic strategies in combination with the conjugative plasmids encoding the engineered pilins, offering a potential solution to the growing problem of antibiotic resistance.

## MATERIALS AND METHODS

### Bacterial strains and culture conditions

The bacterial strains and plasmids used in this study are presented in Table S1. *Escherichia coli* and *Pseudomonas aeruginosa* (PA) strains were cultured at 37°C in Luria-Bertani (LB) (1% tryptone, 1% NaCl, and 0.5% yeast extract) broth or LB plates with 2% bacteriological agar (Acumedia). Ampicillin (50 µg/mL) was used for *E. coli*, while rifampicin (50 µg/mL), carbenicillin (200 µg/mL), and gentamicin (30 µg/mL) were used for PA.

### Preparation of phage lysates

Phage lysates of MP29 and MPK7 were generated by using the plate lysate method, with PAO1 as the host strain, as described elsewhere (7). Lysates of LeviOr01 and PP7 were prepared using PAK cells containing the corresponding cDNA, cultured in 200 mL LB broth for 16 h at 37°C as described previously (6, 13). Phages were precipitated overnight at 4°C with 10% polyethylene glycol (PEG; average molecular weight, 8,000 Da) (Sigma-Aldrich) and 1 M NaCl. The precipitated phages were resuspended in phage buffer [50 mM Tris-HCl (pH 7.5), 10 mM MgSO_4_, and 100 mM NaCl], concentrated by ultracentrifugation at 180,000 × *g* for 6 h and then dissolved in phage buffer (1 mL). Phage titers were determined as plaque forming units (pfu).

### Twitching motility assay

A single colony grown overnight on an LB agar plate was picked with a toothpick and stabbed into the base of a 3-mm-thick 1.5% LB agar plate (19). Following 24 h incubation at 30°C, the twitching zone at the agar-petri dish interface was visualized by staining with 0.1% crystal violet.

### Phage susceptibility assay

Phage susceptibility was assessed via a traditional spotting assay, as described by Choi et al. (20). Serial dilutions (3 µL) of phage lysates were spotted onto the LB agar overlay containing 0.7% agar with 100 µL of PA cells at the late logarithmic phase (OD_600_ of 1.0). Plates were incubated overnight at 37°C.

### Pilin grouping and phylogeny

The *pilA* gene and adjacent sequences, including accessory genes, were amplified by PCR from in-house PA strains grown on LB agar plates. Approximately 1.6 × 10^4^ cells were used as a template using the primer pair, pilA-NF and pilA-CR (Table S2). PCR products were sequenced, and pilin grouping was based on the amplicon size and the pilin (PilA) sequence (21). PilA protein sequences were aligned using Clustal X (ver. 1.83) with default parameters. A phylogenetic tree was constructed using MEGA11 (ver. 11.0.13), employing the neighbor-joining method based on Clustal W alignments (22, 23).

### Pilin expression in the surrogate strain

Pilin switching experiments were performed using the PAK-based surrogate system, which exploited the in-frame *pilA* deletion mutant (Δ*pilA*) of the PAK strain (8). The regions encompassing the accessory gene (*tfpO*) and *pilA* from group I (G1) pilins were PCR-amplified from PA strains using the primer pair, pilA-SLIC_F and pilA-SLIC_R (Table S2). The PCR products were cloned into pUCP18 via sequence and ligation-independent cloning (SLIC) (24), and the resulting plasmids were introduced into the PAK Δ*pilA* by electroporation (Bio-Rad MicroPulser) (20).

### Creation of pilin mutants

Site-directed mutagenesis was employed to generate substitution, in-frame deletion, and insertion mutants at the αβ loop region of PAO1 and PMM23 pilins. Mutagenesis was conducted using four-primer SOEing (splicing by overlap extension) PCR with specific primer pairs (Table S2). Mutant constructs were cloned into pUCP18 via SLIC and then introduced into the PAK Δ*pilA* by electroporation after sequence verification.

### Surface protein assay

Cell surface proteins including flagellins and pilins were isolated using the method described in Chung et al. (19) with some modifications. Bacterial cells were streaked in a grid pattern on LB agar plates containing antibiotics and incubated at 37°C overnight. Cells were softly scraped from the agar surface using a scraper and resuspended in 1 mL of PBS (pH 7.4) per sample. After vortexing vigorously for 3 min, the suspensions were harvested at 15,000 × *g* for 5 min. The supernatant was centrifuged at 15,000 × *g* for an additional 30 min to remove any remaining cells and then transferred to a new microcentrifuge tube. To precipitate the sheared surface proteins, we added 1/10 (vol) of 5 M NaCl and 30% (wt/vol) PEG, and the samples were incubated overnight at 4°C and then pelleted at 15,000 × *g* for 30 min at 4°C. The pellets were resuspended in PBS and electrophoresed on a 15% (wt/vol) SDS-PAGE gel. Proteins were visualized using Coomassie Brilliant Blue R.

### Phage adsorption assay

Phage adsorption was performed by using LeviOr01 lysates following previously established protocols (6, 8). Briefly, the surrogate cells expressing PMM23 pilin mutants were cultured on TS (tryptic soy) agar plates at 37°C for 16 h. Approximately 3 × 10^8^ colony-forming units (cfu) of the cells were then resuspended in 500 µL of TS broth. Phages (10^7^ pfu) were added and incubated with the bacterial cells at 37°C for 5 min to allow adsorption. The phage-cell mixture was centrifuged at 500 × *g* for 10 min at 4°C to separate the adsorbed phages. The supernatant containing unbound phages was recovered, and the pfu were quantified using a plaque assay.

### Structural prediction and comparison

The structure of PAO1 pilin was generated by PyMOL (v. 2.3.0) (PDB code 8TUM), and the structure of PMM23 pilin was predicted using AlphaFold2 (v. 2.3.1). Three models were created, and the top-ranked model (ranked_0.pdb) was selected based on the average pLDDT score. Superimposed pilin structures and key residues in interactions between PP7 MP and PAO1 pilin were visualized using PyMOL (v. 2.3.0).

## Data Availability

Data reported in this study will be shared by the corresponding author upon request, and any additional information required to reanalyze the data reported in this study is available upon request.

## SUPPLEMENTAL MATERIAL

Supplemental figures jvi.01949-24-s0001.pdf Heat map and phylogenetic tree, structural modeling of MP-pilin interaction, and phage absorption to the pilin mutants.

Supplemental tables jvi.01949-24-s0002.pdf Bacterial strains, plasmids, and primers used in this study.

ASM does not own the copyrights to Supplemental Material that may be linked to, or accessed through, an article. The authors have granted ASM a non-exclusive, world-wide license to publish the Supplemental Material files. Please contact the corresponding author directly for reuse.
