# Supplementary material for: Pilin regions that select for the small RNA phages in Pseudomonas aeruginosa type IV pilus
Source: J Virol. 2025 Feb 27;99(4):e01949-24. doi: 10.1128/jvi.01949-24 (PMC11998500; doi:10.1128/jvi.01949-24)
Supplement: Supplemental figures — Heat map and phylogenetic tree, structural modeling of MP-pilin interaction, and phage absorption to the pilin mutants. [file jvi.01949-24-s0001.pdf]

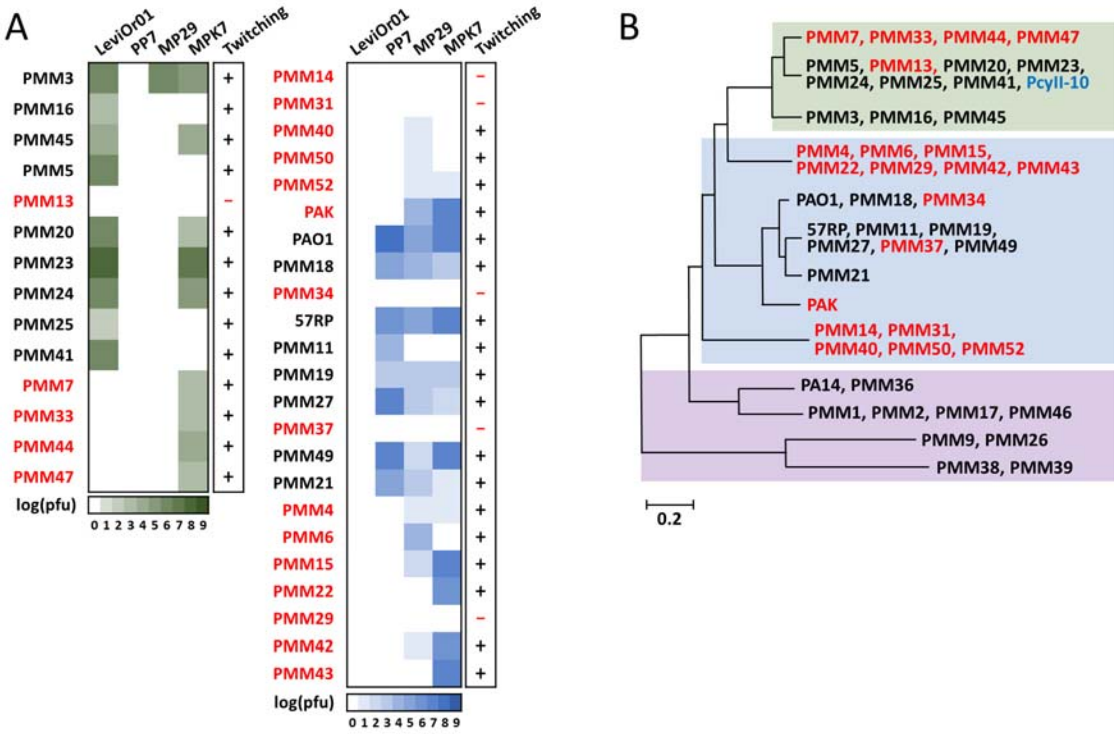

Figure S1. RNA phage plaque formation in some PA strains, related to Figure 1.

A. Heat map of the relative efficiency of plaque formation (EOP) for RNA phages, LeviOr01 and PP7. Color codes represent the relative values of log(pfu) as indicated for the RNA phages (LeviOr01 and PP7) and the DNA phages (MP29 and MPK7). Values represent the average of three biological replicates. PA strains with group **I** (G1) pilin (left) and those with group **II** (G2) pilin (right) are separated for comparison, with either LeviOr01-resistant or PP7-resistant strains designated in red. The capability of twitching motility of each strain is indicated at the right of the heat map: +, twitching-proficient; - (red), twitching-defective.

B. Phylogenetic tree of the 48 TFP pilins. Amino acid sequences of the *pilA*-encoded pilins from Pcy**II**-10 (a known host for LeviOr01, blue) and 47 in-house PA strains were aligned using Clustal W and the neighbor-joining tree has been generated. Ten Group **III** (G3), 23 G2, and 15 G1 pilins are designated in color shades: G3, purple; G2, blue; G1, green. LeviOr01-resistant and PP7-resistant strains are designated in red. The bar scale indicates the pairwise differences among the pilins.

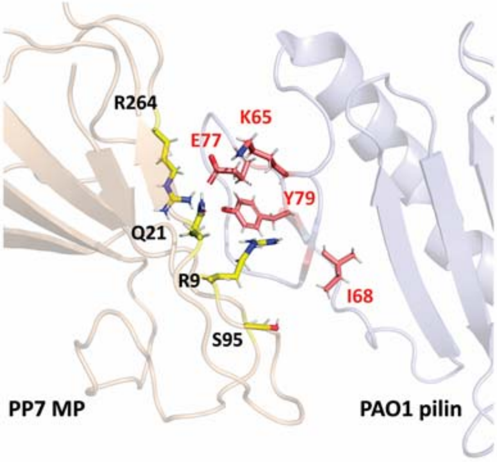

Figure S2. Structural modeling of MP-pilin interaction, related to Figures 2 and 3.
The interaction between PP7 MP and G2 pilin is reconstituted from the structural modeling data (PBD code. 8TUW), which has been modeled using RosettaDock (9). The key amino acids at the minor β-sheet pilus-interacting region of MP (i.e. R9, Q21, S95, and R264) and at the αβ loop region of G2c pilin (K65, I68, E77, and Y79) are suggested to be involved in the electrostatic interaction between MP and pilin.

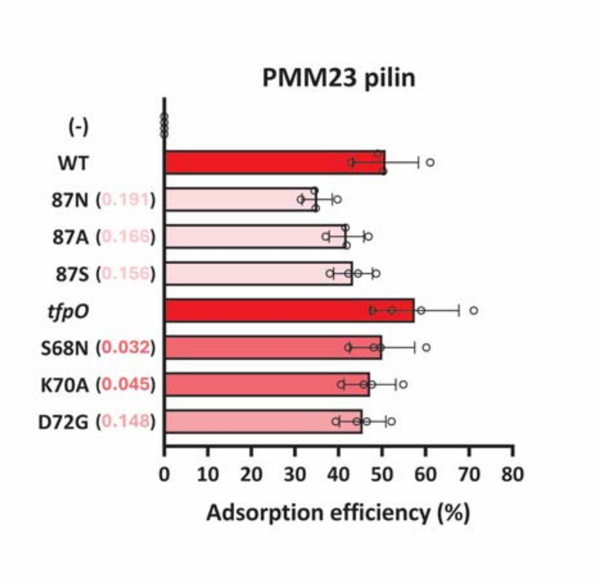

Figure S3. Phage adsorption to the pilin mutants, related to Figure 4.

LeviOr01 phages were incubated with the surrogate strains expressing PMM23 pilin mutants in parallel to the buffer (-) and the wild type (WT) PMM23 pilin as the controls. Unbound phages in the supernatant were measured by plaque assay to calculate the adsorption efficiency. The average values from four biological replicates are shown with the error bars representing standard deviations. The color codes (pink to red) outline the degrees of adsorption efficiency and the RMSD values calculated from AlphaFold3-based structural superimposition of the corresponding mutant pilins with the WT pilin.
