# Supplementary material for: Pilin regions that select for the small RNA phages in Pseudomonas aeruginosa type IV pilus
Source: J Virol. 2025 Feb 27;99(4):e01949-24. doi: 10.1128/jvi.01949-24 (PMC11998500; doi:10.1128/jvi.01949-24)
Supplement: Supplemental tables — Bacterial strains, plasmids, and primers used in this study. [file jvi.01949-24-s0002.pdf]

Table. S1. Bacterial strains and plasmids used in this study.

Strain or plasmid	Relevant characteristics or purpose[a]	Reference or source
Pseudomonas aeruginosa		
PAO1	Wild type laboratory strain	Laboratory collection
PA14	Wild type laboratory strain; Rif^R	Laboratory collection
57RP	Environmental isolate	Eric Déziel
PAK	Wild type laboratory strain	Laboratory collection
PAK *pilA*	PAK with in-frame deletion of *pilA*	Kim et al., 2018
PAK(PP7)	PAK with the chromosomally integrated PP7 cDNA at *att*Tn*7*; Gm^R	Lee et al., 2019
PAK(LeviOr01)	PAK with the chromosomally integrated LeviOr01 cDNA at *att*Tn*7*; Gm^R	Laboratory collection
PMM1-PMM51	Forty-three clinical isolates from various Asian countries	Laboratory collection
Escherichia coli		
DH5α	Multipurpose cloning	Laboratory collection
Phage		
PP7	*Pseudomonas* phage *Pepevirus rubrum*	Laboratory collection
LeviOr01	*Pseudomonas* phage *Pepevirus spumicola*	Laboratory collection
MP29	*Pseudomonas* phage *Casadabnvirus* MP29	Laboratory collection
MPK7	*Pseudomonas* phage *Phikmvvirus* MPK7	Laboratory collection
Plasmids		
pUCP18	General purpose cloning vector; Cb^R	Laboratory collection
pUCP-$pilA_{G1}$	pUCP18 with the pilin regions containing both *tfpO* and *pilA* genes of a G1 pilin; Cb^R	This study
pUCP-$pilA_{PAK}$	pUCP18 with the wild-type PAK pilin; Cb^R	Kim et al., 2018
pUCP-$pilA_{PAO1(WT)}$	pUCP18 with the wild-type PAO1 pilin; Cb^R	Kim et al., 2018
pUCP-$pilA_{PAO1(I68S)}$	pUCP18 with the PAO1 pilin mutant for I68S substitution; Cb^R	This study
pUCP-$pilA_{PAO1(Y79D)}$	pUCP18 with the PAO1 pilin mutant for Y79D substitution; Cb^R	This study

pUCP-pilA$_{PMM23(WT)}$	pUCP18 with the wild-type PMM23 pilin; Cb^R	This study
pUCP-pilA$_{PMM23(87N)}$	pUCP18 with the PMM23 pilin mutant for insertion of N at the 87th position; Cb^R	This study
pUCP-pilA$_{PMM23(87A)}$	pUCP18 with the PMM23 pilin mutant for insertion of A at the 87th position; Cb^R	This study
pUCP-pilA$_{PMM23(87S)}$	pUCP18 with the PMM23 pilin mutant for insertion of S at the 87th position; Cb^R	This study
pUCP-pilA$_{PMM23(\Delta tfpO)}$	pUCP18 with the PMM23 pilin mutant for in-frame deletion of *tfpO*; Cb^R	This study
pUCP-pilA$_{PMM23(S68N)}$	pUCP18 with the PMM23 pilin mutant for S68N substitution; Cb^R	This study
pUCP-pilA$_{PMM23(K70A)}$	pUCP18 with the PMM23 pilin mutant for K70A substitution; Cb^R	This study
pUCP-pilA$_{PMM23(D72G)}$	pUCP18 with the PMM23 pilin mutant for D72G substitution; Cb^R	This study

[a]Rif^R, rifampicin-resistant; Gm^R, gentamicin-resistant; Cb^R, carbenicillin- and ampicillin-resistant.

Table S2. Primers used in this study.

Primer or probe	Relevant characteristics or purpose	Oligonucleotide sequence (5'–3')[a]
pilA-NF	PCR for PilA sequence-based Pilin grouping	CAGCGACAGCTTGTTGCGCTG
pilA-CR	PCR for PilA sequence-based Pilin grouping	CATTACGAATGAGCTGCTCTAC
pilA-SLIC_F	SOEing PCR for *pilA* cloning	CGGTACCCGGGGATCCATTACGAATGAGCTGCTCTAC
pilA-SLIC_R	SOEing PCR for *pilA* cloning	CGACTCTAGAGGATCCAGCGACAGCTTGTTGCGC
O1pilA_I68S_IF	SOEing PCR for PAO1 PilA(I68S) cloning	GGTAGCAAAATTAAA**TC**TGGTACTACTGCTTC
O1pilA_I68S_IR	SOEing PCR for PAO1 PilA(I68S) cloning	GAAGCAGTAGTACCA**GA**TTTAATTTTGCTACC
O1pilA_Y79D_IF	SOEing PCR for PAO1 PilA(Y79D) cloning	CTACTGCGACCGAAACA**G**ATGTCGGCGTCGAGCCG
O1pilA_Y79D_IR	SOEing PCR for PAO1 PilA(Y79D) cloning	CGGCTCGACGCCGACAT**C**TGTTTCGGTCGCAGTAG
23pilA_87N_IF	SOEing PCR for PMM23 PilA(87N) cloning	CTCTGCTTACCGGT**AAC**GACGGTAAGGGGCAGATC
23pilA_87N_IR	SOEing PCR for PMM23 PilA(87N) cloning	GATCTGCCCCTTACCGTC**GTT**ACCGGTAAGCAGAG
23pilA_87A_IF	SOEing PCR for PMM23 PilA(87A) cloning	CTCTGCTTACCGGTG**CAG**ACGGTAAGGGGCAGATC
23pilA_87A_IR	SOEing PCR for PMM23 PilA(87A) cloning	GATCTGCCCCTTACCGT**CTG**CACCGGTAAGCAGAG
23pilA_87S_IF	SOEing PCR for PMM23 PilA(87S) cloning	CTCTGCTTACCGGT**TCC**GACGGTAAGGGGCAGATC
23pilA_87S_IR	SOEing PCR for PMM23 PilA(87S) cloning	GATCTGCCCCTTACCGTC**GGA**ACCGGTAAGCAGAG
23pilA_Δ*tfpO*_IF	SOEing PCR for PMM23 PilA(Δ*tfpO*) cloning	CGGTATTCTCCGGCCTGGGGGGAATATCGGTTG
23pilA_Δ*tfpO*_IR	SOEing PCR for PMM23 PilA(Δ*tfpO*) cloning	CAACCGATATTCCCCCCAGGCCGGAGAATACCG
23pilA_S68N_IF	SOEing PCR for PMM23 PilA(S68N) cloning	GCTCGTTTCCAACGAT**AA**TCCCAAAAACGATGAG
23pilA_S68N_IR	SOEing PCR for PMM23 PilA(S68N) cloning	CTCATCGTTTTTGGGA**TT**ATCGTTGGAAACGAGC
23pilA_K70A_IF	SOEing PCR for PMM23 PilA(K70A) cloning	CCAACGATTCTCCC**GC**AAACGATGAGTATGATC
23pilA_K70A_IR	SOEing PCR for PMM23 PilA(K70A) cloning	GATCATACTCATCGTTT**GC**GGGAGAATCGTTGG
23pilA_D72G_IF	SOEing PCR for PMM23 PilA(D72G) cloning	CGATTCTCCCAAAAACG**G**TGAGTATGATCTTGGC
23pilA_D72G_IR	SOEing PCR for PMM23 PilA(D72G) cloning	GCCAAGATCATACTCA**C**CGTTTTTGGGAGAATCG

[a]Underlining denotes the engineered restriction enzyme sites; the mutated nucleotide residues are boldfaced and underlined

REFERENCES

Kim, E.S., Bae, H.W., Cho, Y.H., 2018. A pilin region affecting host range of the *Pseudomonas aeruginosa* RNA phage, PP7. Front. Microbiol. 9, 247. https://doi.org/10.3389/fmicb.2018.00247.

Lee, J.Y., Ahn, S.J., Park, C., Bae, H.W., Kim, E.S., Cho, Y.H., 2019. Reverse genetic systems for *Pseudomonas aeruginosa* leviphages. Methods. Protoc. 2(1), 22. https://doi.org/10.3390/mps2010022.
